# Local anaesthetics upregulate nitric oxide generation in cord blood and adult human neutrophils

**DOI:** 10.1038/s41598-018-37090-9

**Published:** 2019-01-24

**Authors:** Karolina I. Kulinska, Maria Billert, Krzysztof Sawinski, Katarzyna Czerniak, Michał Gaca, Krzysztof Kusza, Krzysztof W. Nowak, Maria Siemionow, Hanna Billert

**Affiliations:** 1 Department of Experimental Anaesthesiology, Chair of Anaesthesiology and Intensive Therapy, Poznan University of Medical Sciences, 14, Sw. Marii Magdaleny st., 61-861 Poznan, Poland; 2 Department of Animal Physiology and Biochemistry, Poznan University of Life Sciences, 33, Wolynska st., 60-637 Poznan, Poland; 3 Department of Haematology and Bone Marrow Transplantation, Poznan University of Medical Sciences, 82/84, Szamarzewskiego st., 60-569 Poznan, Poland; 4 Clinics of Anaesthesiology in Obstetrics and Gynecology, Chair of Anaesthesiology and Intensive Therapy, Poznan University of Medical Sciences, 33, Polna st., 60-101 Poznan, Poland; 5 Clinics of Anaesthesiology and Intensive Therapy, Chair of Anaesthesiology and Intensive Therapy, Poznan University of Medical Sciences, 49, Przybyszewskiego st., 60-355 Poznan, Poland; 6 University of Illinois at Chicago, Department of Orthopaedics MC 944, 900 South Ashland Avenue, 3356 MCBRB, Chicago, Illinois 60607 USA

## Abstract

Nitric oxide (NO) generation by systemic neonatal neutrophils is not clarified. It is also not known whether local anaesthetics (LAs) transferred to the fetal systemic circulation following maternal epidural blockade may affect this process. In the present study, NO generation was evaluated in neutrophils from cord blood (CB, n = 11) and adult blood (n = 10) following exposure to bupivacaine (0.0005, 0.005, 1 mM), lidocaine (0.002, 0.02, 4 mM) and ropivacaine (0.0007, 0.007, 1.4 mM) using flow cytometry, as well as indirectly by determining nitrite concentrations in cell incubation media. To determine the role of NO synthase (NOS) isoforms in NO generation following exposure to LAs, experiments were repeated in the presence of the NOS inhibitors, *N*^G^-nitro-L-arginine methyl ester and aminoguanidine; in addition, the expression of NOS isoforms was analysed. CB neutrophils produced less NO than adult neutrophils. LAs, especially ropivacaine and lidocaine, stimulated neutrophil NO generation, but in CB neutrophils this effect was negligible at clinically relevant drug concentrations. A mechanism involving NOS activity was responsible for the observed phenomena. In conclusion, LAs are able to upregulate neutrophil NO production, but in neonates this effect is likely to be clinically insignificant.

## Introduction

Neutrophils represent most abundant systemic leukocyte population critical for host defense, especially for the initiation, coordination and resolution of the inflammatory response. These cells produce nitric oxide (NO), a pleiotropic radical gasotransmitter, predominantly in a process of oxidation of L-arginine to L-citrulline and NO, using NADPH and oxygen. The reaction is catalyzed by nitric oxide synthase (NOS)^1^. In neutrophils, all three NOS isoforms (NOS1–3)^2^ are expressed. NOS2 activity quantitatively determines NO generation and is typically referred to as an inducible NOS isoform. However, in resting human neutrophils NOS2 transcript and protein have been found to be constitutively present^3^. Human neutrophils produce relatively small amounts of NO, probably due to epigenetic silencing of *NOS2* expression^4,5^. Remarkably, even nanomolar concentrations of NO have auto- and paracrine effects, significantly modulating neutrophil adhesion, migration, antibacterial activity, the formation of neutrophil extracellular traps (NET) and apoptosis^1,6–8^. Neutrophil NO generation is altered in both chronic and critical conditions and can affect the course of disease^9,10^. For example, in sepsis, NO production by neutrophils stabilizes neutrophil tissue infiltration and then decreases gradually, which corresponds to the resolution of inflammation and survival^10^. Ontogenic aspects of neutrophil NO generation have not been sufficiently clarified. It has been suggested that neutrophil NO generation is impaired in infants and then stabilizes in older children^11^, but to date there is no information as to neutrophil NO generation by fetal and neonatal neutrophils.

Studies addressing the impact of anaesthesia on neutrophil NO generation are scarce^12,13^. Local anaesthetics (LAs), ion channel blockers which temporarily block nociceptive stimulation and also exert biological effects in non-excitable cells, were suggested to stimulate neutrophil NO generation in adult volunteers by only one study^13^. LAs are widely administered in obstetrics for epidural blockades for labour analgesia and anaesthesia and following placental transfer may affect fetal and neonatal circulating cells^14,15^. Previous studies including our own have demonstrated that at clinically relevant concentrations, LAs are able to decrease the activities of neonatal neutrophils critical for host defense, such as chemotaxis and ROS generation^15,16^, closely interlinked with intracellular NO production^1^.

In the present study we compared NO generation in neonatal (cord blood, CB) versus adult circulating neutrophils and investigated the effects of the LAs bupivacaine, lidocaine and ropivacaine on NO generation, specifically with regard to the putative role of NOS in both cell populations studied. Lower NO production was found for CB neutrophils, which corresponded to NOS2 deficiency. In addition, the LA-induced increases in NO generation, especially by ropivacaine and lidocaine, were less pronounced in CB neutrophils. The underlying upregulation of NOS activity and NOS isoform expression varied between neonatal and adult neutrophils.

## Results

Obstetric characteristics and newborn data are summarized in Table 1.

**Table 1:** Obstetric characteristics and the newborn data.

Mother	
Primigravida/multigravida	5/6
Gestational age (weeks)	40 (39–40)
Time (min)	
Rupture of membranes	180 (45–390)
Stage I labour	365 ± 22
Stage II labour	15 (10–40)
Labour analgesia (n)	
None	2
Meperidine	3
Meperidine/Entonox	3
Nalbuphine/Entonox	1
Nalbuphine/TENS/Entonox	1
Nalbuphine/Meperidine	1
Maternal body temperature during II stage of labour (°C)	36.5 ± 0.3
Newborn	
Male/female (n)	6/5
Body mass (g)	3755 ± 761
Placenta weight (g)	669 ± 102
Apgar score 1^st^ min	10 (10–10)
Apgar score 5^th^ min	10 (10–10)
Cord blood gas and pH post labour	
- Umbilical artery	
pH_a_	7.26 ± 0.10
P_a_o_2_ (mmHg)	26.9 ± 7.3
P_a_co_2_ (mmHg)	51.0 ± 12.8
BE_a_	–5.5 ± 2.1
- Umbilical vein	
pH_v_	7.30 ± 0.07
P_v_o_2_ (mmHg)	27.1 ± 6.8
P_v_co_2_ (mmHg)	42.2 (35.3–48.7)
BE_v_	−5.4 ± 1.9

Categorized data are presented as numbers, data fitting normal distribution are presented as means ± standard deviation, data that don’t fit normal distribution – as median (interquartile range).

TENS - Transcutaneous electrical nerve stimulation, BE – base excess.

### NO production in cord blood versus adult neutrophils

**Figure 1:**
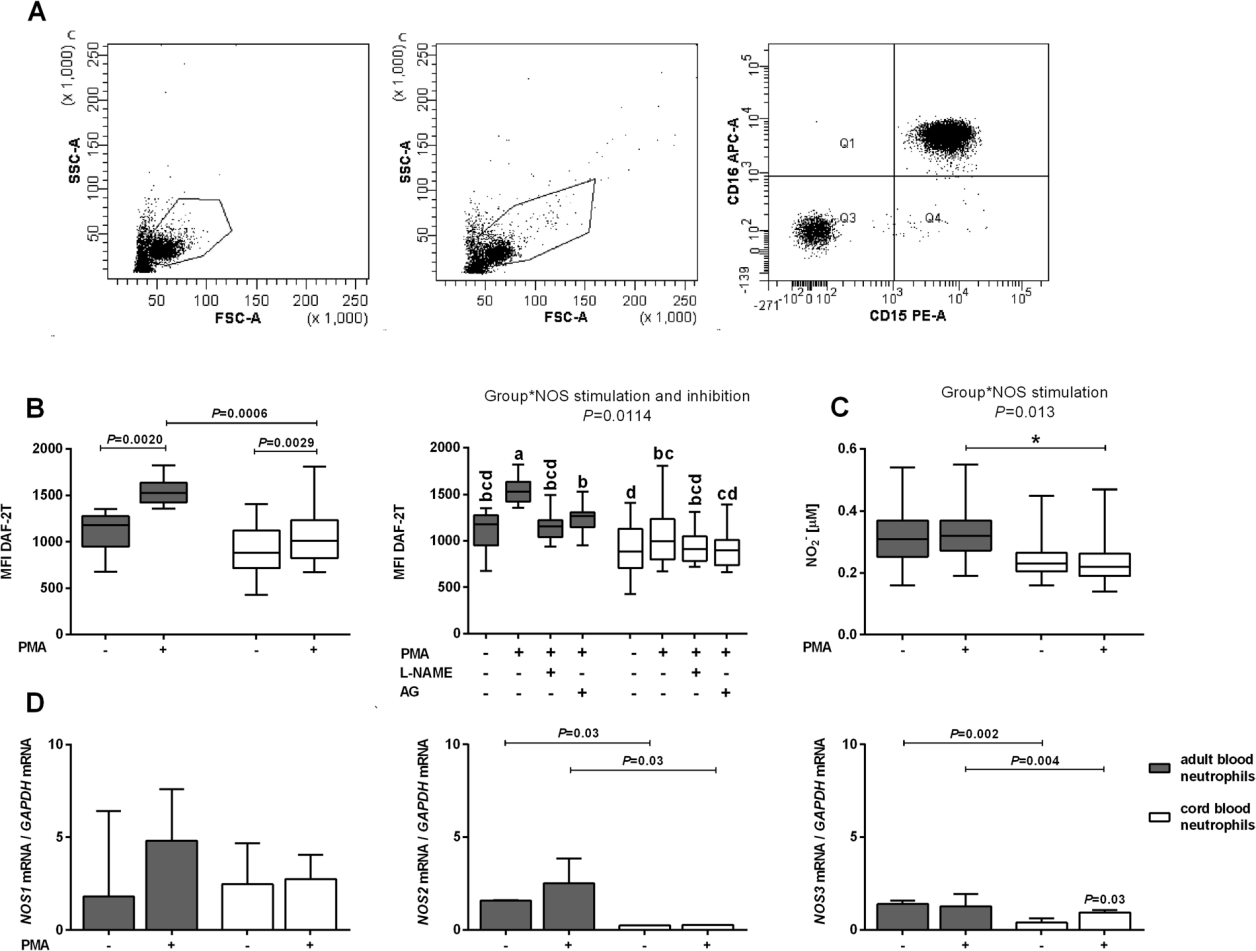
Cytometric neutrophil identification, nitric oxide (NO) generation and expression of NO synthase (NOS) isoforms in cord (white bars) and adult (gray bars) peripheral blood neutrophils. (**A**) Representative dot plot of adult (left graph) and cord blood neutrophils (middle graph) identified by forward and side scatter characteristics (FSC and SSC, respectively) and FL-2/FL-4 fluorescence (right graph). Neutrophils were incubated with fluorochrome-conjugated monoclonal antibodies (i.e. anti-CD15–phycoerythrin (PE) and anti-CD16–allophycocyanin (APC)) and gated as FSC^high^ SSC^high^, and CD16^high^CD15^high^ (Q2 gate; 97.2%). 10 000 events were collected. (**B**) Intracellular NO production and (**C**) nitrite concentrations in incubation media of unstimulated and phorbol myristate acetate (PMA)-stimulated cord blood neutrophils (n = 11) and adult blood neutrophils (n = 10). Data are shown as box plots (median, interquartile range and min-max). (**B**) Intracellular NO production was determined by flow cytometry as described in the Materials and Methods and expressed as mean fluorescence intensity of triazolofluorescein (MFI DAF-2T). Left graph: Data were analysed by Wilcoxon and Mann–Whitney U-tests. Right graph: The functional role of NOS in the intracellular NO generation, as indicated by the graphical representation of a significant interaction between the factors ‘group’ (cord or adult blood neutrophils) and ‘NOS stimulation and inhibition’ (unstimulated neutrophils, PMA-stimulated neutrophils, PMA-stimulated neutrophils incubated with *N*^G^-nitro-L-arginine methyl ester (L-NAME) or aminoguanidine (AG)) determined by multifactorial ANOVA/*post-hoc* Tukey tests. Comparisons are presented using abcd notation - means with the same letter are not significantly different from each other (*P* > 0.05). Further details are summarized in Table S1. (**C**) Nitrite concentrations in neutrophil incubation media are shown by graphical representation of significant interactions between the factors ‘group’ (cord or adult blood neutrophils) and ‘NOS stimulation’ (unstimulated neutrophils, PMA-stimulated neutrophils) determined by multifactorial ANOVA/*post-hoc* Tukey tests, *P < 0.05. Nitrite concentrations were determined using ozone-based chemiluminescence as described in the Materials and Methods. Further details are summarized in Table S2. (**D**) Median (interquartile range) *NOS1*, *NOS2*, *NOS3* gene expression in cord (n = 6) and adult blood (n = 6) neutrophils incubated in the absence or presence of 0.97 μM PMA. Total RNA was isolated using TRI-Reagent and *NOS1, NOS2, NOS3* expression was evaluated using real-time quantitative polymerase chain reaction. Wilcoxon and Mann–Whitney *U*-tests.

Cytometric neutrophil identification is shown in Fig. 1A.

Phorbol myristate acetate (PMA) stimulation resulted in an increase in intracellular NO production, as measured by flow cytometry, in both adult and CB neutrophils (*P* = 0.0020 and *P* = 0.0029 compared with unstimulated cells, respectively; Fig. 1B, left graph). PMA-stimulated NO generation was significantly lower in CB than adult neutrophils (*P* = 0.0006; Fig. 1B, left graph). Lower PMA-stimulated NO production in CB neutrophils was confirmed by the significant interaction between the ‘group’ and ‘NOS stimulation and inhibition’ factors (see Materials and Methods for details; *P* = 0.0114; Fig. 1B, right graph; see Table S1 available as Supplementary Material to this paper).

The NOS inhibitors *N*^G^-nitro-l-arginine methyl ester (l-NAME) and aminoguanidine (AG) entirely abolished the stimulatory effect of PMA on NO production in adult neutrophils only. The effects of l-NAME and AG were comparable (Fig. 1B, right graph).

Nitrite concentrations were lower in the incubation media of PMA-stimulated CB than adult neutrophils, with a significant interaction identified between the ‘group’ and ‘NOS stimulation’ factors (*P* = 0.013; Fig. 1C; see Table S2).

Both CB and adult blood neutrophils expressed all three NOS isoforms (Fig. 1D). *NOS2* and *NOS3* expression was significantly lower in stimulated and unstimulated cord blood neutrophils than in adult blood neutrophils (*P* = 0.03 and *P* = 0.03, for *NOS2* in unstimulated and PMA-stimulated neutrophils, respectively; *P* = 0.002 and *P* = 0.004 for *NOS3* in unstimulated and PMA-stimulated neutrophils, respectively). There were no significant differences in *NOS1* expression between the two cell populations (i.e. adult and cord blood) studied. *NOS3* expression was enhanced in stimulated compared with unstimulated CB neutrophils (*P* = 0.03).

### Effects of LAs on NO generation in cord blood and adult neutrophils

According to multifactorial analysis of variance (ANOVA), enhancement of NO production by neutrophils incubated with LAs depends on the type of anaesthetic, its concentration, the cell population studied and NOS stimulation or inhibition, as indicated by significant interactions between the factors ‘anaesthetic’, ‘concentration category’, ‘group’, and ‘NOS stimulation and inhibition’ (see Materials and Methods). Raw data of the LAs effects on intracellular NO generation (cytometric data) and nitrite concentrations in neutrophil incubation media were presented in Tables S3 and S4, respectively.

#### Effects of LA type and concentration

**Figure 2:**
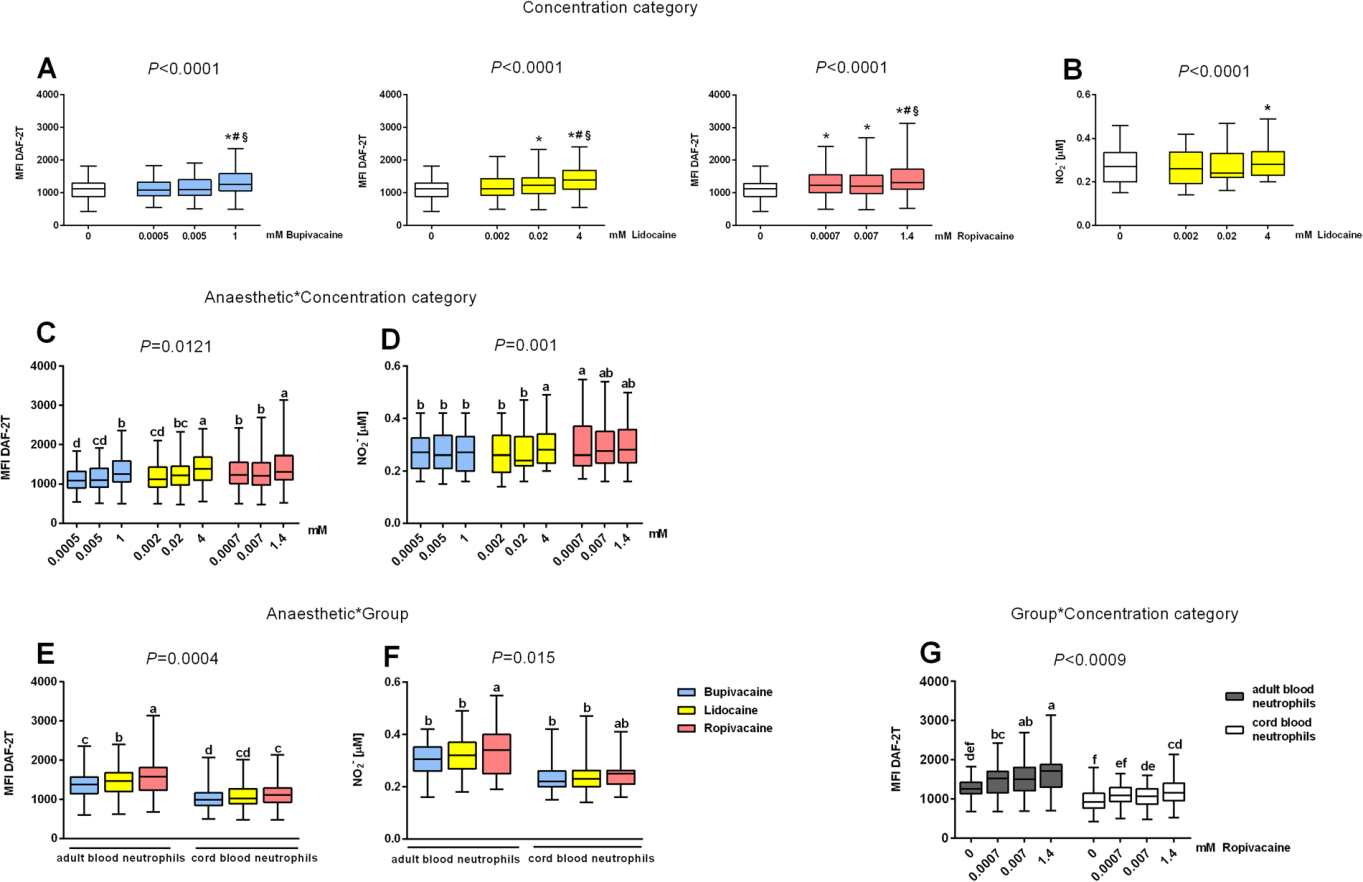
Effects of bupivacaine, lidocaine and ropivacaine on the neutrophil nitric oxide (NO) generation, (**A**–**D**) overall and (**E**–**G**) separately in cord and adult blood neutrophils as shown by graphical representations of significant interactions determined by multifactorial ANOVA between the factors of ‘anaesthetic’ (type of anaesthetic), ‘concentration category’ (concentration categorized into three levels: lowest, middle and highest) and ‘group’ (cord or adult blood neutrophils). (**A**,**B**) Significant concentration-dependent overall effects of the local anaesthetics (LAs; significant factor ‘concentration category’) on (**A**) intracellular NO generation and (**B**) nitrite levels in incubation media (significant effect only for lidocaine). *P < 0.05 vs. control (0), ^#^*P* < 0.05 vs. the lowest concertation, ^§^*P* < 0.05 vs. middle concertation. (**C**,**D**) Effects of LA concentrations on (**C**) intracellular neutrophil NO production and (**D**) nitrite concentration in the incubation media as shown by significant interactions between the ‘anaesthetic’ and ‘concentration category’ factors. (**E**,**F**) Differences in the effects of LAs on cord and adult blood neutrophils showing significant interactions between the ‘group’ and ‘anaesthetic’ factors on intracellular NO generation (**E**) and nitrite concentration in incubation media (**F**). (**G**) Differences in the effects of the applied concentrations of ropivacaine on intracellular NO generation in cord and adult blood neutrophils showing a significant interaction between the ‘group’ and ‘concentration category’ factors. NO production was determined by flow cytometry and expressed as mean fluorescence intensity of triazolofluorescein (MFI DAF-2T); nitrite concentrations were measured using ozone-based chemiluminescence as described in the Materials and Methods. Data are displayed in box plots (median, interquartile range and min-max). Datasets are compared using abcd notation; means with the same letter are not significantly different from each other (*P* > 0.05; multifactorial ANOVA followed by Tukey test). Further details are summarized in Tables S2, S5 and S6.

All anaesthetics stimulated intracellular neutrophil NO generation in a concentration-dependent manner, as shown by flow cytometry. Overall, bupivacaine enhanced intracellular neutrophil NO production only at the highest concentration tested (*P* < 0.0001; Fig. 2A; Table S6), lidocaine enhanced NO production at the highest (maximum effect) and middle concentrations tested (*P* < 0.0001), whereas ropivacaine enhanced NO production at all concentrations tested (*P* < 0.0001). Indirect assessment of NO production, namely measurement of nitrite in cell incubation media, revealed that only lidocaine at the highest concentration tested had a significant effect (*P* < 0.0001; Fig. 2B; Table S6).

The stimulatory effects of LAs on neutrophil NO generation were collectively summarized using multifactorial ANOVA comparative models. Significant interactions were found between the ‘anaesthetic’ and ‘concentration category’ factors for both direct cytometric assessment of NO production (*P* = 0.0121; Fig. 2C; Table S5), and nitrite measurements in neutrophil cell incubation media (*P* = 0.001; Fig. 2D; Table S2). NO production was higher for neutrophils incubated with the highest concentrations of lidocaine and ropivacaine than for neutrophils incubated with the highest concentration of bupivacaine. Determining nitrite concentrations in the incubation medium as an indirect measure of NO production revealed that the effect of the highest concentration of lidocaine was greater than that of bupivacaine. At the lowest, clinically relevant LA concentrations, incubation of cells with ropivacaine resulted in the highest NO production compared with lidocaine and bupivacaine, with the effects of the latter two LAs being comparable (Fig. 2C,D; Tables S2, S5).

**Figure 3:**
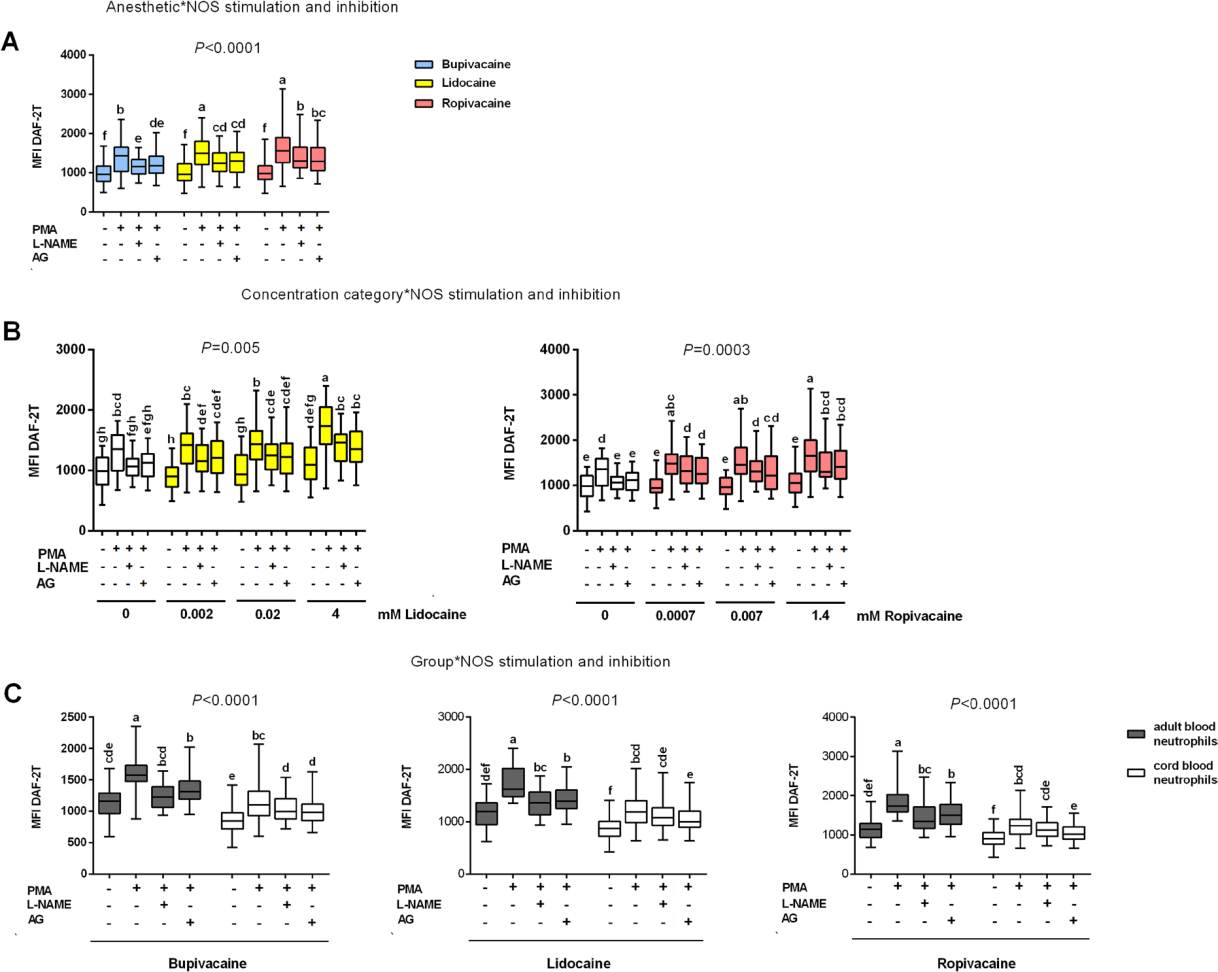
Functional role of nitric oxide synthase (NOS) in the effects of local anaesthetics (LAs) on intracellular neutrophil nitric oxide (NO) generation, (**A**,**B**) overall and (**C**) in cord (n = 11) and adult blood neutrophils (n = 10) as shown by graphical representations of significant interactions determined by multifactorial ANOVA between the factors of ‘anaesthetic’ (type of anaesthetic), ‘concentration category’ (concentration categorized into three levels: lowest, middle and highest), ‘group’ (cord or adult blood neutrophils) and ‘NOS stimulation and inhibition’ (unstimulated neutrophils, phorbol myristate acetate (PMA)-stimulated neutrophils, PMA-stimulated neutrophils incubated with *N*^G^-nitro-l-arginine methyl ester (l-NAME) or aminoguanidine (AG)). (**A**,**B**) Overall effect of NOS inhibition on intracellular NO generation in unstimulated and PMA-stimulated neutrophils exposed to: (**A**) the three LAs, showing a significant interaction between ‘anaesthetic’ and ‘NOS stimulation and inhibition’; and (**B**) lidocaine (left graph), and ropivacaine (right graph), showing a significant interaction between ‘concentration category’ and ‘NOS stimulation and inhibition’. (**C**) Differences in intracellular NO generation between unstimulated and PMA-stimulated adult and cord blood neutrophils exposed to bupivacaine, lidocaine and ropivacaine in the presence of NOS inhibitors showing a significant interaction between ‘group’ and ‘NOS stimulation and inhibition’. NO production was determined by flow cytometry and expressed as mean fluorescence intensity of triazolofluorescein (MFI DAF-2T); nitrite concentrations were measured using ozone-based chemiluminescence as described in the Materials and Methods. Data are displayed in box plots (median, interquartile range and min-max). Datasets are compared using abcd notation; means with the same letter are not significantly different from each other (*P* > 0.05; multifactorial ANOVA followed by Tukey test). Further details are summarized in Tables S5, S6.

In PMA-stimulated neutrophils, the effects of lidocaine and ropivacaine on NO production were comparable and more pronounced than those of bupivacaine, as demonstrated by the significant interaction between the ‘anaesthetic’ and ‘NOS stimulation and inhibition’ factors (*P* < 0.0001; Fig. 3A; Table S5).

In contrast with bupivacaine, lidocaine further enhanced intracellular NO production in PMA-stimulated neutrophils, but only at the highest concentration tested, as demonstrated by a significant interaction between the ‘concentration category’ and ‘NOS stimulation and inhibition’ factors (*P* = 0.005; Fig. 3B; Table S6). All concentrations of ropivacaine used increased NO production even further in PMA-stimulated compared with unstimulated neutrophils (*P* = 0.0003; Fig. 3B, right graph; Table S6).

#### Effects of group

Overall, CB neutrophils exposed to bupivacaine, lidocaine and ropivacaine produced less NO than adult cells, as indicated by significant interaction between the ‘anaesthetic’ and ‘group’ factors (*P* = 0.0004; Fig. 2E). Incubation of neutrophils with ropivacaine resulted in higher NO generation than that seen following incubation with bupivacaine. In adult neutrophils, the following rank order of potency was observed: ropivacaine > lidocaine > bupivacaine. In PMA-stimulated neutrophils, higher intracellular NO generation in adult than CB neutrophils was confirmed for each LA tested by significant interactions between the ‘group’ and ‘NOS stimulation and inhibition’ factors (*P* < 0.0001; Fig. 3C).

Intracellular NO generation was lower in CB than adult neutrophils for all concentrations of ropivacaine tested, as demonstrated by significant interactions between the ‘group’ and ‘concentration category’ factors (*P* < 0.0009; Fig. 2G; Table S6). Ropivacaine failed to stimulate NO production in CB neutrophils at the lowest, clinically relevant concentration. For the other two LAs, the interaction between the ‘group’ and ‘concentration category’ factors was not significant.

Using nitrite as an indirect measure of NO production revealed a significant interaction between the ‘group’ and ‘anaesthetic’ factors (*P* = 0.015; Fig. 2F; Table S2). Nitrite concentrations in the incubation media of adult neutrophils were higher for neutrophils exposed to ropivacaine than for those exposed to bupivacaine and lidocaine. However, there were no significant differences in nitrite concentrations in the incubation media of CB neutrophils exposed to different LAs, or compared with adult cells.

#### Role of NOS

Overall, the NOS inhibitors l-NAME and AG partly and comparably reduced the stimulatory effects of PMA and LAs on intracellular NO generation by neutrophils (significant interaction between the ‘anaesthetic’ and ‘NOS stimulation and inhibition’ factors; *P* < 0.0001; Fig. 3A; Table S6). As indicated by significant interactions between the ‘concentration category’ and ‘NOS stimulation and inhibition’ factors for both lidocaine (*P* = 0.005) and ropivacaine (*P* = 0.0003), NOS inhibitors only partly suppressed NO generation following exposure of neutrophils to particular concentrations of these LAs (Fig. 3B; Table S6).

Among the neutrophil populations studied, the effects of NOS inhibitors in samples exposed to particular LAs were similar and incomplete (significant interactions between the ‘group’ and ‘NOS stimulation and inhibition’ factors; *P* < 0.0001; Fig. 3C; Table S6). Only in PMA-stimulated adult neutrophils incubated with bupivacaine did l-NAME completely abolish the stimulatory effects of the LA on NO generation. In CB PMA-stimulated neutrophils exposed to lidocaine and ropivacaine, the LA-induced increase in NO production was partly reduced only by AG.

**Figure 4:**
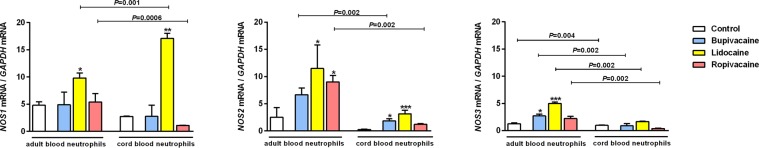
Expression of the three nitric oxide synthase (NOS) isoforms (*NOS1, NOS2* and *NOS3*) in the phorbol myristate acetate (PMA)-stimulated cord (n = 6) and adult blood neutrophils (n = 6) exposed to local anaesthetics. *NOS1, NOS2* and *NOS3* expression was evaluated using real-time quantitative polymerase chain reaction as described in Materials and Methods. Data are the median and interquartile range. Friedman/*post-hoc* Dunn tests; *P < 0.05, **P < 0.01, ***P < 0.001; Mann-Whitney U-test.

Exposure to the highest concentrations of LAs resulted in the expression of different NOS isoforms in CB and adult PMA-stimulated neutrophils (Fig. 4). In CB neutrophils, bupivacaine and lidocaine enhanced *NOS2* expression (*P* < 0.05 and *P* < 0.001, respectively). The stimulatory effect of lidocaine on *NOS1* expression in these cells was most pronounced, with a greater than sixfold increase in expression of *NOS1* mRNA (*P* < 0.01). *NOS3* expression in CB cells was not affected by LAs. Ropivacaine did not affect the expression of any NOS isoform in this neutrophil population. In adult neutrophils, lidocaine enhanced the expression of all NOS isoforms (*P* < 0.05 for both *NOS1* and *NOS2*; *P* < 0.001 for *NOS3*), whereas ropivacaine enhanced the expression of *NOS2* only (*P* < 0.05) and bupivacaine only enhanced the expression of *NOS3* (*P* < 0.05).

Of note, following exposure to lidocaine, *NOS1* expression was approximately twofold higher in CB than adult neutrophils (*P* = 0.001). For all other NOS isoforms, expression was always lower in CB than adult neutrophils following exposure to LAs. *NOS2* and *NOS3* expression was lower in CB than adult neutrophils incubated with bupivacaine (*P* = 0.002 for both isoforms) and the expression of all NOS isoforms was lower in CB than adult neutrophils following exposure to ropivacaine (*P* = 0.0006 for *NOS1, P* = 0.002 for *NOS2* and *NOS3*).

### Cytotoxic effects of LAs

**Figure 5:**
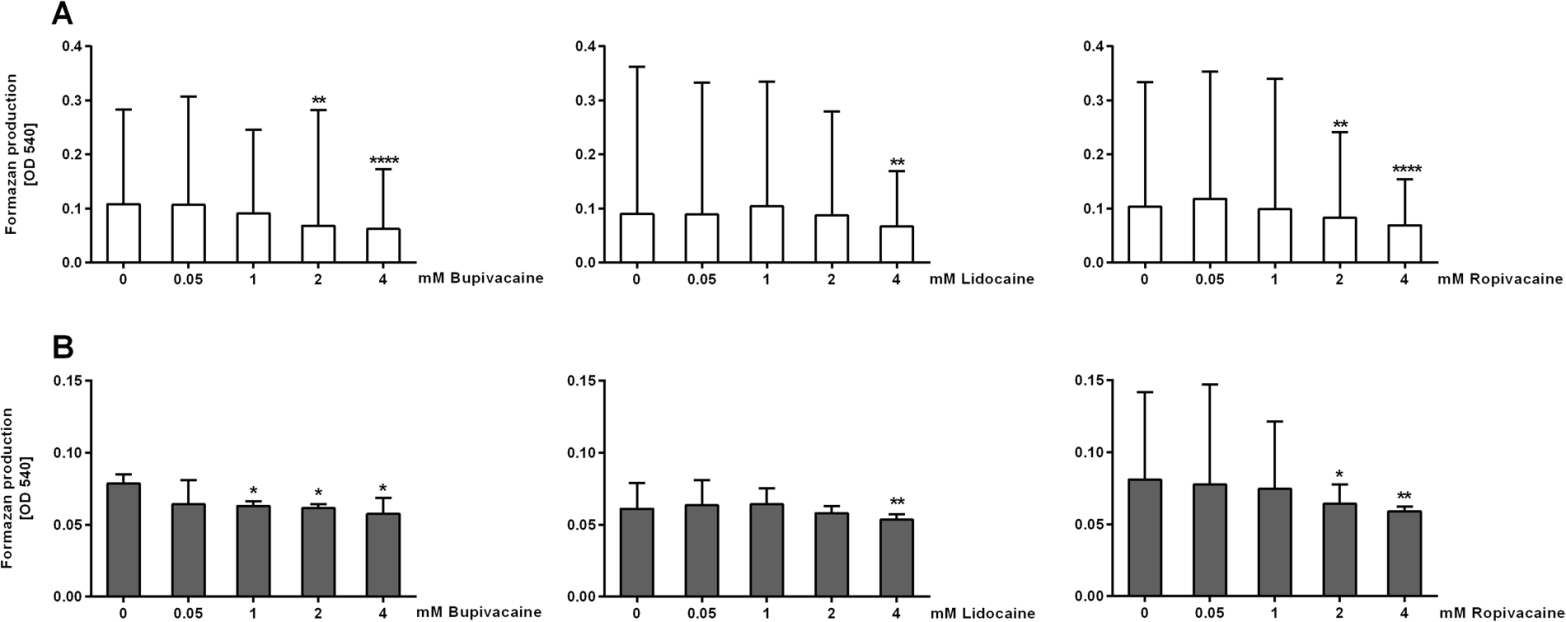
Cytotoxicity of local anaesthetics in (**A**) cord (n = 4; white bars) and (**B**) adult peripheral blood neutrophils (n = 3; gray bars). Cytotoxicity was assessed by 3-(4,5-dimethylthiazol-2-yl)-2,5-diphenyltetrazolium bromide (MTT) test as described in Materials and Methods. Measurements were done in sextuplicates. Data are the median and interquartile range. Friedman/*post-hoc* Dunn test; *P < 0.05, **P < 0.01, ****P < 0.0001.

As assessed by the 3-(4,5-dimethylthiazol-2-yl)-2,5-diphenyltetrazolium bromide (MTT) assay, until the highest concentration applied in the present study, bupivacaine did not render cytotoxic effects in adult neutrophils (*P* < 0.05, Fig. 5); at this concentration CB neutrophils were still viable. In both neutrophil populations, lidocaine exerted cytotoxic effects at the highest concentration only (*P* < 0.01), and ropivacaine at the concentrations of 2 and 4 mM.

## Discussion

The present study showed that: (1) PMA-stimulated CB neutrophils produce less NO than adult cells, and that NO production in CB neutrophils is mediated via a NOS2-independent mechanism; (2) LAs increase NO generation by neutrophils depending on the type of anaesthetic and concentration; (3) following exposure to LAs, CB neutrophils produce less NO than adult neutrophils; and (4) LAs affect NO generation by neutrophils by differentially upregulating NOS activity and NOS isoform expression in CB and adult neutrophils.

PMA stimulation resulted in a significant increase in intracellular NO generation in both adult and neonatal neutrophil populations. Our finding that PMA-stimulated CB neutrophils generate lower amounts of NO than adult cells was confirmed using both methodological approaches in the present study, namely direct cytometric measurement and indirect assessment using nitrite concentrations in the neutrophil incubation media as an index of NO production. Upon PMA stimulation, intracellular NO generation in adult neutrophils increased by approximately one-fourth of the initial values, remarkably close to the results obtained in LPS-stimulated neutrophils^17^. Negligible levels of *NOS2* transcripts in neonatal neutrophils and no suppression of intracellular NO generation by NOS inhibitors indicated that, in CB neutrophils, NO was not generated by NOS2 activity, unlike in adult cells. Similar results were reported by Tsui *et al*.^18^ who found lower Shiga toxin-stimulated intracellular neutrophil NO generation accompanied by impaired *NOS2* mRNA expression in infants compared with older children and adults. In line with their findings, in the present study unstimulated intracellular NO generation was comparable between CB and adult neutrophils, possibly due to low basal NO production^18^. As an alternative to the NOS-dependent mechanism, which, in adults, is responsible for approximately two-thirds of neutrophil NO production, another possible source of NO may be a non-enzymatic reaction between H_2_O_2_ and arginine^9,19^. Lower *NOS2* expression was also reported in cord versus adult peripheral blood progenitor cells^20^. Remarkably, CB cells are characterized by hypermethylation of the interferon (IFN)-γ promoter, functionally interlinked with *NOS2* in humans^21^. Lower NO generation by CB neutrophils may also be the result of the effects of constituents present in higher concentrations in cord than adult blood, such as asymmetric dimethylarginine (ADMA), an endogenous NOS inhibitor^22^ and adenosine, which inhibits NO production and CD11b expression^23^. In addition, the reduced bioavailability of the substrate l-arginine due to increased activity of arginase I, needs to be taken into consideration^24^. Upregulated *NOS3* expression in stimulated CB neutrophils could be explained by the relatively hyperoxic conditions after birth compared with the intrauterine milieu, as reported for rat neonatal haemopoietic cells^25^. Remarkably, lower NO generation may correspond to the suppression of a number of functions associated with NO signalling in CB neutrophils^1,6–8,26^.

In line with previous suggestions^13^, we demonstrated that LAs can increase neutrophil NO generation overall and in PMA-stimulated neutrophils depending on the concentration of the agent. Notably, stimulatory effects of LAs on NO generation were reported yet only in glioma cells; in macrophages and endothelial cells these agents decreased NO synthesis^27,28^. In this study, we also showed that the exposed to LAs CB neutrophils produced significantly lower intracellular amounts of NO than adult cells, as reflected by a significant interaction between the ‘anaesthetic’ and ‘group’ factors.

Using an overall approach of multifactorial ANOVA we established that anaesthetic concentration was a significant factor generally determining the increase in neutrophil NO production, independently of the cell population studied. Significant interactions between the factors ‘anaesthetic’ and ‘concentration category’ were noted for the results obtained by both the cytometric and nitrite methodological approaches. Regarding intracellular NO generation, the maximum effects elicited by the highest concentrations of lidocaine and ropivacaine were comparable and more pronounced than those elicited by bupivacaine. Using the nitrite approach, this was found to be true only for lidocaine, in line with findings reported in rat glioma cells^29^. At the lowest concentration, ropivacaine was the most effective agent. LAs did not render cytotoxic effects until the highest concentration applied. A causal link between increased intracellular NO generation and apoptosis in human neutrophils was recently reported^8^. Remarkably, measurements of nitrite concentrations in the culture media did not allow to detect differences in neutrophil NO generation between CB and adult neutrophils. The discrepancies in results between the direct and indirect methodological approaches could be explained by differences in the nature of intracellular NO generation and nitrite formation in the incubate. Because auto-oxidation is too slow to be of biological significance, the process of nitrite formation requires additional enzymatic oxidative activity that, in the lung epithelium, has been suggested to be a specific membrane-associated haem-containing oxidase^30^. Thus, our data show that the interpretation of nitrite concertation in neutrophil incubation media as an index of intracellular NO production should be made with caution.

Regarding intracellular NO generation, we could observe an overall enhancement of PMA stimulation effects by LAs, specifically by lidocaine at the highest concentration and ropivacaine at the all applied concentrations, as indicated by significant interactions between anaesthetic concentration and the NOS stimulation or inhibition process. Upregulation of intracellular neutrophil NO formation by ropivacaine, not yet assessed in this regard, deserves emphasizing. Ropivacaine, the only chiral agent of the LAs tested, is structurally similar to bupivacaine but has lower lipophilicity (i.e., similar to lidocaine) and these distinct properties should be considered as possibly significant for the observed differences in drug activity.

Studies suggest comparative reactivity of neonatal and adult neutrophils to anti-inflammatory compounds^31^. Regarding LAs, in a previous investigation we found only minor differences in the effects of bupivacaine and lidocaine on neutrophil ROS generation^15^. In the present study, lower intracellular NO synthesis by the pre-exposed to LAs and PMA-stimulated cord blood neutrophils could be attributed to the aforementioned weaker effect of PMA stimulation. However, significant differences between neonatal and adult neutrophils independently of the stimulation effect were also noted. Specifically, as demonstrated by the interaction between the ‘group’ and ‘concentration category’ factors, in cells incubated with ropivacaine, independently of whether they were stimulated or not, neonatal neutrophils produced less NO at every studied concentration. This finding may be interpreted as a stimulatory effect of ropivacaine on intracellular NO generation, apparently weaker in neonatal neutrophil population. Correspondingly, ropivacaine failed to upregulate *NOS2* expression in these cells, which might at least partly explain this phenomenon. Moreover, CB neutrophils incubated with ropivacaine produced more NO that was produced after exposure of cells to bupivacaine, which, of the three LAs, has the highest membrane affinity. Distinct lipid profiles and lower fluidity of the membranes of CB cells may be of importance in explaining these findings^32^. Remarkably, in contrast with adult neutrophils, no stimulatory effect of ropivacaine on NO production by neutrophils at the lowest, clinically relevant concentration tested implies minor clinical significance of this phenomenon. In view of the preferable use of ropivacaine in perinatal settings and possible effector implications, such as decreased neutrophil adhesion, which is of concern in neonatal infection^26^, this finding appears to be good news.

In adult neutrophils, ropivacaine increased NO generation to a greater degree than lidocaine and bupivacaine. Overall, as well as in adult neutrophils specifically, increases in intracellular NO generation following exposure to ropivacaine were seen at all concentrations used. This finding may be of importance in adult perioperative settings, where the agent is widely used in regional techniques for postoperative pain control. Ropivacaine was demonstrated to inhibit neutrophil adhesion in association with NO production^33^, which, under conditions of simultaneous NADPH oxidase (NOX) activation, is able to decrease integrin expression^6^.

Trying to explain the role of NOS activity for the observed in this study stimulating effects of LAs on neutrophil NO generation, NOS inhibitors were used and the effects of LAs on the expression of NOS isoforms were investigated. Comparable effects of the non-specific NOS inhibitor l-NAME and the NOS2-specific inhibitor AG confirmed the importance of upregulation of the NOS2 isoform in the quantitative increase in NO generation, in line with previous suggestions by Mamiya *et al*. based on experiments with canavanine, a plant-derived arginine analogue^13^. Conversely, inhibition of Gαq-proteins by LAs was reported to decrease NOS2 expression in endotoxin-stimulated murine macrophages^27^.

We have demonstrated in the present study that LAs are able to increase neutrophil *NOS* expression. Lidocaine had the most pronounced effect, stimulating all *NOS* isoforms in adult neutrophils and the *NOS1* and *NOS2* isoforms in CB neutrophils. In addition, lidocaine was the only anaesthetic that upregulated *NOS1* expression in both adult and CB neutrophils. Remarkably, *NOS1* stabilizes NOS2 activity and is important for sepsis survival and bacterial clearance^34^. In the present study, lidocaine increased *NOS1* mRNA transcripts in CB neutrophils more than sixfold; of note, in these cells *NOS1* expression was significantly higher, nearly twofold greater, than in adult neutrophils. This surprising phenomenon could possibly be attributed to high oestrogen concentrations in CB during labour^35^. In neutrophils, NOS1 is under strong control of oestrogens, which, conversely, increase transient receptor potential vanilloid 1 activity, a lidocaine target that interacts with *NOS1*, as demonstrated in skeletal muscle cells^36,37^. However, in neutrophils lidocaine was reported to preferentially (compared with bupivacaine and ropivacaine) induce mitochondrial depolarization, which, in neurons, resulted in NOS1 activation^38,39^.

In adult neutrophils, *NOS2* expression was increased following exposure to lidocaine and ropivacaine, which, in contrast with bupivacaine, exhibit demethylating properties^40^. Because human NOS isoforms are under strict epigenetic control^4,5^, epigenetic desuppression of NOS could underlie the stimulation effect, and this warrants further investigation. In case of ropivacaine, a pure *S*(−)enantiomer, increased NOS affinity to chiral ligands depending on substrate and cofactor interactions may be of importance^41^.

LAs failed to modulate cell membrane-associated *NOS3* expression in CB neutrophils at all, which may be attributed to the distinct membrane structure of these cells^32^. Remarkably, in contrast with adults neutrophils, ropivacaine did not affect the expression of any NOS isoforms in CB neutrophils. As already mentioned, this is in addition to the distinct differences in NO production between the neonatal and adult cells. Conversely, the increase in NOS expression in CB neutrophils was partly reduced by AG, suggesting a role for NOS2 activation in these cells. Remarkably, L-NAME only abolished the stimulatory effect in adult neutrophils exposed to bupivacaine, suggesting the contribution of isoforms other than NOS2.

There are some other factors that could possibly modulate upregulation of NOS isoforms by LAs observed in this study. Due to ROS scavenging properties, LAs could attenuate ROS-mediated suppression of NOS activity and the bioavailability of its cofactors, likely to occur upon PMA stimulation^15,42^. LAs are also able to inhibit potassium channels physiologically connected with neutrophil NO generation^43^ which decrease NO bioavailability^44^. In kidney channelopathies, hypokalaemia was associated with decreased expression of G_αq_-proteins and concurrent *NOS3* upregulation in neutrophils^45^. In this study, *NOS3* expression was significantly increased in adult neutrophils exposed to bupivacaine and lidocaine.

The present study has some limitations. First, *in vitro* studies do not reflect the real effect of the agents tested in clinical settings. Second, for ethical reasons, most mothers received opioids for labour pain control, potentially suppressing NOS activity^46^. Third, using density gradient separation to isolate neutrophils means that we could not avoid some monocyte contamination, which could have affected *NOS* expression results^47^. Fourth, the methods used to assess neutrophil NO generation have certain shortcomings. With regard to the direct cytometric method to determine intracellular NO, using 4,5-diaminofuorescein diacetate solution (DAF-2DA) is associated with concerns regarding fluorochrome specificity; the measurement covers not only NO, but also reactive nitrogen species, in part^48^. Nitrite estimation in the culture medium is an indirect method and the involvement of additional oxidase activity in the generation of nitrite should be taken into consideration^30^.

Taken together, we examined not previously studied NO generation by neonatal neutrophils, and demonstrated that upon activation with phorbol ester these cells produce NO, albeit in significantly lower amounts than adult neutrophils. Moreover, we showed that this phenomenon was accompanied by lower neonatal NOS expression (NOS2 and NOS3 isoforms). As NO production in neutrophils is connected with a number of cellular effector functions critical for host defence, this finding may indicate hitherto unconsidered molecular link explaining a nature of neutrophil impairment in the newborn. Physiological significance of a relative deficiency of NOS expression and NO generation in CB neutrophils may be related to protection against the cytotoxic actions of reactive nitrogen and oxygen species (i.e., peroxynitrite). Assessing the effects of LAs on NO generation in neonatal and adult neutrophils we showed that these two cell populations reacted more intensely to PMA stimulation while pre-exposed to LAs. However, this phenomenon was less evident in neonatal cells regarding all agents tested. Differences between neonatal and adult cell populations were most expressed in neutrophils exposed to ropivacaine, an anaesthetic preferentially used for various regional techniques including obstetrics, and not yet examined in regard of NO generation. The strongest ability of ropivacaine to stimulate NO production independently of the neutrophil population studied (as shown by multifactorial ANOVA), and specifically in adult cell population, is a novel interesting finding. It may be of significance in surgical adult patients, in whom, in contrast to neonates, the increasing action was detected at all concentrations tested. Its direct connection with impaired integrin expression and neutrophil adhesion needs to be confirmed in experimental and clinical settings. Eventually, the strength of the present study was that we showed an upregulation of NOS isoforms as a molecular background of increased NO generation by neutrophils exposed to LAs. NOS2 expression was increased following incubation with lidocaine and ropivacaine in adult neutrophils, whereas in CB cells ropivacaine did not upregulate any *NOS* isoform. Regarding *NOS1*, a regulatory NOS isoform, lidocaine exerted a surprisingly strong stimulatory effect in both studied cell populations, especially pronounced in CB neutrophils. Lidocaine is the only LA administered intravenously for several clinical indications; interestingly, it was shown to be able, in a combination with adenosine and magnesium to provide tissue protection and control severe infections even instead antibiotics^49^. The property of lidocaine to preferentially upregulate neutrophil *NOS1* isoform is a novel finding and deserves further investigation.

To the best of our knowledge, this is the first report comparing the effects of three different LAs on NO generation in cord blood and adult neutrophils and, as such, should contribute to a better understanding of the effects of LAs in clinical settings.

## Materials and Methods

The present study was approved by the Bioethics Committee of Poznan University of Medical Sciences (669/12, 660/15) and performed in accordance with relevant guidelines. Mothers of newborns and volunteers provided written informed consent before donation of blood samples. Exclusion criteria involved regional methods for labour pain control, maternal disease, smoking and medication. Samples (18 mL) of CB were drawn immediately after cord clamping from healthy, full-term newborns (n = 11), whereas peripheral blood samples were collected from healthy, non-smoking male donors aged 20–40 years (n = 10) into heparinized vacutainers (S-Monovette; Sarstedt, Nümbrecht, Germany) and processed within 1 h.

### Neutrophil separation

Neutrophils were separated by density gradient centrifugation. For neonatal neutrophils, our own modification of a classical protocol was applied due to different characteristics of CB (altered cell composition and lower plasma viscosity). Briefly, cord blood was pre-incubated with 1.5% dextran solution (molecular mass 500 000 Da; Lab Empire, Rzeszow, Poland) and for removal of erythrocytes was sedimented at room temperature (RT) for 45 min. To improve neutrophil separation from mononuclear cells, the leucocyte-rich cord blood plasma was layered over Gradisol G (density 1.119 g/cm^3^; 3 mL; Aqua-Med, Lublin, Poland) and Gradisol L (density 1.077 g/cm^3^; 1.5 mL; Aqua-Med) and was centrifuged at 400 *g* at RT for 30 min.

Adult blood was layered over Gradisol G and centrifuged at 400 *g* at RT for 30 min. Contaminating erythrocytes were removed by hypotonic water lysis. Then, both neutrophil populations were washed twice with phosphate-buffered saline (PBS; Sigma-Aldrich, St Louis, MO, USA), centrifuged at 300 *g* at RT for 10 min and then resuspended in Ca^2+^- and Mg^2+^-free Hank’s balanced salt solution (HBSS; Institute of Immunology and Experimental Therapy, Polish Academy of Sciences, Wroclaw, Poland). Neutrophil viability was >98%, as assessed by Trypan blue (Sigma-Aldrich) dye exclusion, and purity, as assessed microscopically, was >88%. After counting, 1.2 × 10^6^ neutrophils were seeded onto cell culture plates and incubated with LAs (see below) until cytometric analysis.

### Neutrophil incubation with LAs

Water stock solutions of LAs were stored at 4 °C. Prior to experiments, 10xPBS was added to the stock solutions to obtain final equipotent LA concentrations of 0.0005, 0.005 and 1 mM bupivacaine (Bupivacaine hydrochloride monohydrate; Sigma-Aldrich), 0.002, 0.020, 0.2 and 4 mM lidocaine (Lidocaine hydrochloride monohydrate; Sigma-Aldrich) and 0.0007, 0.007 and 1.4 mM ropivacaine (Ropivacaine hydrochloride monohydrate; Sigma-Aldrich). The concentrations used were adopted to allow comparisons regarding clinical potency: bupivacaine is three to four times more potent than lidocaine, and ropivacaine has 0.5–0.7 potency of bupivacaine. The lowest concentrations of bupivacaine and ropivacaine used corresponded to those observed in cord blood during maternal neuraxial blockade^14^ and were followed by 10 and 200 times higher ones. The highest concentration was used as a reference value often tested in studies addressing the influence of LAs on various aspects of neutrophil biology. Neutrophils were incubated with LAs under standard conditions (37 °C, 5% CO_2_) for 2 h.

### Flow cytometry

Neutrophils were identified by flow cytometry (FACS CantoII; BD Biosciences, San Jose, CA, USA) based on forward (FSC) versus side scatter (SSC) characteristics and the expression of CD15 and CD16 antigens.

After incubation with fluorochrome-conjugated monoclonal antibodies (i.e. anti-CD15–phycoerythrin (PE) and anti-CD16–allophycocyanin (APC; BD Biosciences) in the dark at 4 °C for 10 min, neutrophils were washed with Cell Wash (BD Biosciences) and centrifuged at 300 *g* at 4 °C for 10 min. Debris and lymphocytes were excluded based on FSC versus SSC analysis. Neutrophils were gated as CD15^high^ CD16^high^. In all, 10 000 events were collected.

After treatment with LAs, neutrophil samples were pre-incubated with vehicle or NOS inhibitors, namely non-specific L-NAME (1 mM; Sigma-Aldrich) and NOS2-specific aminoguanidine (AG; 1 mM; Sigma-Aldrich) at 37 °C for 30 min before being stimulated with 0.97 μM PMA (Sigma-Aldrich) at 37 °C for 15 min. AG binds covalently to NOS2 protein and to the haem residue at the active site, not disrupting the integrity of the haem porphyrin ring, and inhibits NOS2 with little effect on NOS1 and NOS3^50^. Concentrations of NOS inhibitors used were based on the literature^1^ and our own preliminary experiments. Stimulation with PMA instead of inflammatory factors was used because neonatal neutrophils have lower endotoxin binding properties and lower Toll-like receptor (TLR) 4 downstream signalling activity^51^. In the next step, for the detection of intracellular NO generation, neutrophils were loaded with a cell permeable analogue of DAF-2, DAF-2DA, that is intracellularly converted to DAF-2 which traps NO to create fluorescent DAF-2T^48^. The procedure was performed according to an assay for measuring free NO and NOS activity in living cells, Fluorimetric Nitric Oxide Synthase Detection System (Sigma Aldrich). Briefly, after PMA stimulation neutrophils were washed, and 0.5 µM DAF-2DA (Sigma-Aldrich), 0.1 mM L-arginine (Sigma-Aldrich), 1 mM NOS inhibitor, and buffer were added. Then, cells were incubated in dark at RT for 2 h. 10 min before the end of the incubation, propidium iodide (PI, 0.5 µg/10^6^ cells; Lab-Empire) was added to exclude dead cells.

DAF-2T+ cells were gated as 10 000 live (PI-negative) cells with high fluorescence in the FL1/DAF-2T fluorescein isothiocyanate (FITC) channel. Gates with high DAF-2T fluorescence (FL1 channel) were set compared with non-fluorescent control. The MFI for the 10 000 acquired events was calculated using FACS Diva Software (BD Biosciences).

### Nitrite concentrations

Nitrite concentrations in the neutrophil incubation media were determined using ozone-dependent chemiluminescence (NOA280i; GE Analytical Instruments, Boulder, CO, USA). The method is based on nitrite reduction to NO which reacts with an ozone molecule forming nitrogen dioxide in an activated state. Then, the compound reverts to a lower energy state emitting light detected by a photomultiplier. Intensity of chemiluminescence is proportional to the nitrite concentration in the measured sample. The measurements were performed using NOA analyser according to the manufacturer’s protocol (Sievers Nitric Oxide Analyzer NOA 280i Operation and Maintenance Manual, USA, 2006). Briefly, 90 µl of culture media was injected in duplicate into the purge vessel containing Nitrite Reducing Agent (5 ml glacial acetic acid, 50 mg potassium iodide, 2 ml deionized water) and infused with nitrogen. NO released was carried to the analyser by the nitrogen gas stream. Then, the chemiluminescence signals were measured. Nitrite concentrations were calculated using calibration curve of different KNO_2_ concentrations. The sensitivity of this assay for liquid samples is 1 pM.

### Expression of NOS isoforms

The expression of NOS isoforms was analysed by quantitative real-time polymerase chain reaction (PCR). Total RNA was isolated from neutrophils (0.5 × 10^6^ cells) using TRI-Reagent (Lab-Empire). The purity and quantity of isolated RNA were determined using a NanoDrop (ThermoScientific, Wilmington, DE, USA). Then, 0.5 µg total RNA was reverse-transcribed using the Transcriptor First Strand cDNA Synthesis Kit (Roche Diagnostics, Rotkreuz, Switzerland). *NOS1, NOS2, NOS3* and the *GAPDH* reference gene were amplified using specific primers for gene transcripts, as follows: NM_000620.4 for the NOS1 isoform; NM_000625.4 for the NOS2 isoform; NM_000603.4 for the NOS3 isoform; and NM_001289745.1 for human *GAPDH*. The primers used to analyse gene expression were as follows: human *GAPDH*, 5′-CGCTCTCTGCTCCTCCTGTT-3′ (forward) and 5′-CCATGGTGTCTGAGCGATGT-3′ (reverse); *NOS1*, 5′-GAGCCAGACAAACCAAAGAAGTAC-3′ (forward) and 5′-GCGCTGGATGGCTTTGAG-3′ (reverse); *NOS2*, 5′-CATTCAGATCCCCAAGCTCTACA-3′ (forward) and 5′-TGCCGAGATTTGAGCCTCAT-3′ (reverse); and *NOS3*, 5′-GGGCAGCCTCACTCCTGTT-3′ (forward) and 5′-ACGGCGTTGGCCACTT-3′ (reverse). Real-time PCR was performed using EvaGreen qPCR master mix (Solis BioDyne, Tartu, Estonia). Relative gene expression was determined using the ΔΔCt method. The expression of target genes was normalized against that of the *GAPDH* reference gene and then analysed using a QuantStudio 12 K Flex Real-Time System (Life Technologies, Grand Island, NY, USA).

#### MTT assay

MTT assay was performed in isolated cord and adult blood neutrophils exposed to different concentrations of LAs at standard conditions for 2 h. Following incubation, neutrophils were seeded into 96-well plates (1.5 × 10^5^ cells/well), 12 µL of MTT reagent (5 mg/mL) was added, and the incubation was continued for the next 3 h. Then, cells were centrifuged and intracellular formazan crystals were dissolved in DMSO. Absorbance was measured using microplate reader (Bio-Tek, USA) at 540 nm.

### Statistical analysis

Data were analysed using Statistica version 12 (StatSoft, Tulsa, OK, USA) and JMP Pro (jmp-11.0) 32 bit 11.0.0. (SAS Institute, Cary, NC, USA). Graphs were drawn using GraphPad Prism 6.0 (GraphPad Software, Inc., La Jolla, CA, USA).

We did not perform *a priori* power analysis assuming sample size based on previous reports and our own experience^13,15^. Data were tested for normal distribution by the Shapiro–Wilk test. Univariate non-parametric analysis (because some data were not normally distributed) was used for comparisons of MFI DAF-2T between control cord and adult blood neutrophils, as well as for comparisons of *NOS* isoform expression in control versus LA-exposed neutrophils and cytotoxicity data (Wilcoxon signed-rank test, Friedman test with *post hoc* Dunn test and the Mann–Whitney *U*-test). Multifactorial ANOVA with *post hoc* Tukey test was used for analyses of the effects of LAs on intracellular NO generation and nitrite concentrations in the incubation media. This approach, which is preferred if the effect of one factor cannot be separated from those of other ones, allows assessment of fixed (experimental conditions) and random (intergroup variability) factors. The following independent factors and interactions between them were considered: ‘anaesthetic’ (i.e. bupivacaine, lidocaine, ropivacaine), ‘concentration category’ (i.e. LA concentrations were categorized according to potency as lowest, middle and highest), ‘group’ (i.e. CB or adult blood neutrophils) and ‘NOS stimulation and inhibition’ (i.e. unstimulated neutrophils, PMA-stimulated neutrophils, PMA-stimulated neutrophils incubated with l-NAME, PMA-stimulated neutrophils incubated with AG (for nitrite estimations ‘NOS stimulation’ only, i.e. unstimulated neutrophils, PMA-stimulated neutrophils). Significant factor effects and interactions are shown graphically. To show differences between data sets, exact *P* values, asterisks or abcd notation were used. Letter notation is typically applied in multifactorial ANOVA graphs and allows comparisons of every single data set with any other one, according to Tukey *post-hoc* test estimations. Significance was set as two-sided *P* < 0.05.

## Supplementary information

Supplementary Data

## Data Availability

All data generated or analysed during this study are included in this published article (and its Supplementary Information files).

## References

[1] Sethi S, Dikshit M (2000). Modulation of polymorphonuclear leukocytes function by nitric oxide. Thromb Res..

[2] Chen LY, Mehta JL (1996). Variable effects of L-arginine analogs on L-arginine-nitric oxide pathway in human neutrophils and platelets may relate to different nitric oxide synthase isoforms. J Pharmacol Exp Ther..

[3] Jyoti A (2014). Interaction of inducible nitric oxide synthase with rac2 regulates reactive oxygen and nitrogen species generation in the human neutrophil phagosomes: implication in microbial killing. Antioxid Redox Signal..

[4] Gross TJ (2014). Epigenetic silencing of the human NOS2 gene: rethinking the role of nitric oxide in human macrophage inflammatory responses. J Immunol..

[5] Buzzo CL (2017). Epigenetic regulation of nitric oxide synthase 2, inducible (NOS2) by NLRC4 inflammasomes involves PARP1 cleavage. Sci Rep..

[6] Bhopale VM, Yang M, Yu K, Thom SR (2015). Factors Associated with Nitric Oxide-mediated β2 Integrin Inhibition of Neutrophils. J Biol Chem..

[7] Lim MB, Kuiper JW, Katchky A, Goldberg H, Glogauer M (2011). Rac2 is required for the formation of neutrophil extracellular traps. J Leukoc Biol..

[8] Dubey M (2016). Nitric oxide-mediated apoptosis of neutrophils through caspase-8 and caspase-3-dependent mechanism. Cell Death Dis..

[9] Abrantes DC (2015). Diminished nitric oxide generation from neutrophils suppresses platelet activation in chronic renal failure. Mol Cell Biochem..

[10] Santos SS (2012). Generation of nitric oxide and reactive oxygen species by neutrophils and monocytes from septic patients and association with outcomes. Shock..

[11] Shiga M, Nishina K, Mikawa K, Obara H (2001). The effects of lidocaine on nitric oxide production from an activated murine macrophage cell line. Anesth Analg..

[12] González-Correa JA (2008). Effects of propofol on the leukocyte nitric oxide pathway: *in vitro* and *ex vivo* studies in surgical patients. Naunyn Schmiedebergs Arch Pharmacol..

[13] Mamiya K, Tomoda MK, Edashige K, Ueda W, Manabe M (1995). Local anesthetics enhance nitric oxide production by human peripheral neutrophils. Physiol Chem Phys Med NMR..

[14] Ala-Kokko TI (1997). Feto-maternal distribution of ropivacaine and bupivacaine after epidural administration for cesarean section. Int J Obstet Anesth..

[15] Billert H, Czerniak K, Bednarek E, Kulińska K (2016). Effects of local anesthetics on the respiratory burst of cord blood neutrophils *in vitro*. Pediatr Res..

[16] Birle A (2015). Neutrophil chemotaxis in cord blood of term and preterm neonates is reduced in preterm neonates and influenced by the mode of delivery and anaesthesia. PLoS One.

[17] Ren X, Ding Y, Lu N (2016). Nitrite attenuated peroxynitrite and hypochlorite generation in activated neutrophils. Eur J Pharmacol..

[18] Tsuji S (2012). Production of nitric oxide is lower in Shiga toxin-stimulated neutrophils of infants compared to those of children or adults. Tohoku J Exp Med..

[19] Nagase S (1997). A novel nonenzymatic pathway for the generation of nitric oxide by the reaction of hydrogen peroxide and D- or L-arginine. Biochem Biophys Res Commun..

[20] Muscari C (2007). Different expression of NOS isoforms in early endothelial progenitor cells derived from peripheral and cord blood. J Cell Biochem..

[21] Rico D, Vaquerizas JM, Dopazo H, Boscá L (2007). Identification of conserved domains in the promoter regions of nitric oxide synthase 2: implications for the species-specific transcription and evolutionary differences. BMC Genomics..

[22] von Leitner EC (2011). Pathogenic cycle between the endogenous nitric oxide synthase inhibitor asymmetrical dimethylarginine and the leukocyte-derived hemoprotein myeloperoxidase. Circulation..

[23] Hou PC (2012). Different modulating effects of adenosine on neonatal and adult polymorphonuclear leukocytes. Scientific World Journal..

[24] Kropf P (2007). Arginase activity mediates reversible T cell hyporesponsiveness in human pregnancy. Eur J Immunol..

[25] Marconi GD (2014). Postnatal hyperoxia exposure differentially affects hepatocytes and liver haemopoietic cells in newborn rats. PloS One..

[26] Lawrence SM, Corriden R, Nizet V (2017). Age-Appropriate Functions and Dysfunctions of the Neonatal Neutrophil. Front Pediatr..

[27] Kuo PC, Schroeder RA, Bartlett ST (1997). Endotoxin-mediated synthesis of nitric oxide is dependent on Gq protein signal transduction. Surgery..

[28] Takaishi K, Kitahata H, Kawahito S (2013). Local anesthetics inhibit nitric oxide production and l-arginine uptake in cultured bovine aortic endothelial cells. Eur J Pharmacol..

[29] Feinstein DL (2001). Local anesthetics potentiate nitric oxide synthase type 2 expression in rat glial cells. J Neurosurg Anesthesiol..

[30] Zhao XJ (2013). Mechanisms for cellular NO oxidation and nitrite formation in lung epithelial cells. Free Radic Biol Med..

[31] Craciun EM, Altfelder F, Kuss N, Poeschl J, Ruef P (2013). Anti-inflammatory effects of selected drugs on activated neonatal and adult neutrophils. Scand J Clin Lab Invest..

[32] Yasui K (1990). An increase in polymorphonuclear leucocyte chemotaxis accompanied by a change in the membrane fluidity with age during childhood. Clin Exp Immunol..

[33] Zhu X, Tan Z, Chen J, Zhu M, Xu Y (2010). Effects of ropivacaine on adhesion molecule CD11b expression and function in human neutrophils. Int Immunopharmacol..

[34] Cui X (2007). Neuronal nitric oxide synthase deficiency decreases survival in bacterial peritonitis and sepsis. Intensive Care Med..

[35] Wilson EA, Finn AE, Rayburn W, Jawad MJ (1979). Corticosteroid-binding globulin and estrogens in maternal and cord blood. Am J Obstet Gynecol..

[36] Molero L (2002). Expression of estrogen receptor subtypes and neuronal nitric oxide synthase in neutrophils from women and men: regulation by estrogen. Cardiovasc Res..

[37] Ito N, Ruegg UT, Kudo A, Miyagoe-Suzuki Y, Takeda S (2013). Activation of calcium signaling through Trpv1 by nNOS and peroxynitrite as a key trigger of skeletal muscle hypertrophy. Nat Med..

[38] Kawasaki C, Kawasaki T, Ogata M, Sata T, Chaudry IH (2010). Lidocaine enhances apoptosis and suppresses mitochondrial functions of human neutrophil *in vitro*. J Trauma..

[39] Katakam PV (2016). Depolarization of mitochondria in neurons promotes activation of nitric oxide synthase and generation of nitric oxide. Am J Physiol Heart Circ Physiol..

[40] Lirk P, Hollmann MW, Fleischer M, Weber NC, Fiegl H (2014). Lidocaine and ropivacaine, but not bupivacaine, demethylate deoxyribonucleic acid in breast cancer cells *in vitro*. Br J Anaesth..

[41] Nakano K, Sagami I, Daff S, Shimizu T (1998). Chiral recognition at the heme active site of nitric oxide synthase is markedly enhanced by L-arginine and 5,6,7,8-tetrahydrobiopterin. Biochem Biophys Res Commun..

[42] Singh AK (2016). High oxidative stress adversely affects NFκB mediated induction of inducible nitric oxide synthase in human neutrophils: Implications in chronic myeloid leukemia. Nitric Oxide..

[43] Patel S, Vemula J, Konikkat S, Barthwal MK, Dikshit M (2009). Ion channel modulators mediated alterations in NO-induced free radical generation and neutrophil membrane potential. Free Radic Res..

[44] Gaete PS, Lillo MA, Ardiles NM, Pérez FR, Figueroa XF (2012). Ca2+-activated K+ channels of small and intermediate conductance control eNOS activation through NAD(P)H oxidase. Free Radic Biol Med..

[45] Calò L (2001). Abnormalities of Gq-mediated cell signaling in Bartter and Gitelman syndromes. Kidney Int..

[46] Kampa M (2001). Opioids are non-competitive inhibitors of nitric oxide synthase in T47D human breast cancer cells. Cell Death Differ..

[47] Saluja R (2011). Molecular and biochemical characterization of nitric oxide synthase isoforms and their intracellular distribution in human peripheral blood mononuclear cells. Biochim Biophys Acta..

[48] Räthel TR, Leikert JF, Vollmar AM, Dirsch VM (2003). Application of 4,5-diaminofluorescein to reliably measure nitric oxide released from endothelial cells *in vitro*. Biol Proced Online..

[49] Dobson GP, Letson HL (2016). Adenosine, lidocaine, and Mg2 + (ALM): From cardiac surgery to combat casualty care–Teaching old drugs new tricks. J Trauma Acute Care Surg..

[50] Bryk R, Wolff DJ (1998). Mechanism of inducible nitric oxide synthase inactivation by aminoguanidine and L-N6-(1-iminoethyl)lysine. Biochemistry..

[51] Fragiadakis GK (2016). Mapping the Fetomaternal Peripheral Immune System at Term Pregnancy. J Immunol..

