# Supplementary material for: Local anaesthetics upregulate nitric oxide generation in cord blood and adult human neutrophils
Source: Sci Rep. 2019 Jan 24;9:569. doi: 10.1038/s41598-018-37090-9 (PMC6346062; doi:10.1038/s41598-018-37090-9)
Supplement: Supplementary file 1 — Supplementary Data [file 41598_2018_37090_MOESM1_ESM.docx]

Local anaesthetics upregulate nitric oxide generation in cord blood and adult human neutrophils

Karolina I. Kulińska, Maria Billert, Krzysztof Sawiński, Katarzyna Czerniak, Michał Gaca, Krzysztof Kusza, Krzysztof W. Nowak, Maria Siemionow, Hanna Billert

Supplementary Material

Table S1. Intracellular nitric oxide production in cord blood and adult neutrophils. Multifactorial ANOVA main effects and interactions between the factors: ‘group’ - cord blood neutrophils, adult blood neutrophils; ‘NOS stimulation and inhibition’ – neutrophils unstimulated, PMA-stimulated, PMA-stimulated and incubated with L-NAME, PMA-stimulated and incubated with AG.

DF, degrees of freedom; DF error, the denominator degrees of freedom; F, the ratio of the factor variance to the error variance. * denotes an interaction between the factors. Significance at *P* ˂ 0.05.

|  |  | DF | DF error | F | *P*-value |
| --- | --- | --- | --- | --- | --- |
| Factors | Group | 1 | 19 | 13.881 | 0.0014 |
|  | NOS stimulation and inhibition | 3 | 57 | 19.975 | <0.0001 |
|  |  |  |  |  |  |
| Interaction | Group*NOS stimulation and inhibition | 3 | 57 | 4.028 | 0.0114 |

Table S2. Nitrite concentrations in incubation media of neutrophils exposed to LAs - a comparative model of multifactorial ANOVA. Main effects and interactions between the assessed factors: ‘anaesthetic’ – bupivacaine, lidocaine, ropivacaine; ‘concentration category’ – concentrations of LAs categorized by their potency: the lowest, middle, the highest; ‘group’ - cord blood neutrophils, adult blood neutrophils; ‘NOS stimulation’ - neutrophils unstimulated, PMA-stimulated.

DF, degrees of freedom; DF error, the denominator degrees of freedom; F, the ratio of the factor variance to the error variance. * denotes an interaction between the factors. Significance at *P* ˂ 0.05.

|  |  | DF | DF error | F | *P*-value |
| --- | --- | --- | --- | --- | --- |
| Factors | Group | 1 | 17.03 | 5.758 | 0.028 |
|  | Anaesthetic | 2 | 274.1 | 13.428 | <0.0001 |
| Interaction | Group*anaesthetic | 2 | 274.1 | 4.294 | 0.015 |
|  |  |  |  |  |  |
| Factor | Concentration category | 2 | 274 | 3.649 | 0.027 |
| Interactions | Group*concentration category | 2 | 274 | 0.904 | 0.406 |
|  | Anaesthetic*concentration category | 4 | 274 | 4.752 | 0.001 |
|  | Group*anaesthetic*concentration category | 4 | 274 | 0.193 | 0.942 |
|  |  |  |  |  |  |
| Factor | NOS stimulation | 1 | 274.1 | 0.403 | 0.526 |
| Interactions | Group*NOS stimulation | 1 | 274.1 | 6.271 | 0.013 |
|  | Anaesthetic*NOS stimulation | 2 | 274.1 | 0.267 | 0.766 |
|  | Group*anaesthetic*NOS stimulation | 2 | 274.1 | 0.447 | 0.640 |
|  | Concentration category*NOS stimulation | 2 | 274 | 0.329 | 0.720 |
|  | Group*concentration category*NOS stimulation | 2 | 274 | 1.448 | 0.237 |
|  | Anaesthetic*concentration category*NOS stimulation | 4 | 274 | 0.466 | 0.761 |
|  | Group*anaesthetic*concentration category*NOS stimulation | 4 | 274 | 0.330 | 0.858 |

.

Table S3. Mean values of fluorescence intensity of triazolofluorescein (MFI DAF-2T) of adult (n=10) and cord blood neutrophils (n=11) exposed to LAs.

|  | Bupivacaine (mM) | | | | | | | | | | | | | | | | | | |
| --- | --- | --- | --- | --- | --- | --- | --- | --- | --- | --- | --- | --- | --- | --- | --- | --- | --- | --- | --- |
|  | 0 | | | |  | 0.0005 | | | |  | 0.005 | | | |  | 1 | | | |
|  | us. | PMA | PMA | PMA |  | us. | PMA | PMA | PMA |  | us. | PMA | PMA | PMA |  | us. | PMA | PMA | PMA |
|  |  |  | L-NAME | AG |  |  |  | L-NAME | AG |  |  |  | L-NAME | AG |  |  |  | L-NAME | AG |
| Adult blood neutrophils | 1113  ±216 | 1542  ±155 | 1154  ±157 | 1239  ±150 |  | 1018  ±215 | 1581  ±139 | 1221  ±169 | 1308  ±193 |  | 1089  ±245 | 1587  ±166 | 1239  ±173 | 1366  ±212 |  | 1350  ±298 | 1731  ±381 | 1361  ±217 | 1447  ±301 |
| Cord blood neutrophils | 887  ±268 | 1079  ±319 | 966  ±187 | 930  ±219 |  | 806  ±170 | 1108  ±308 | 997  ±198 | 979  ±221 |  | 803  ±186 | 1114  ±313 | 1011  ±191 | 995  ±233 |  | 981  ±236 | 1291  ±433 | 1187  ±205 | 1154  ±270 |
|  | Lidocaine (mM) | | | | | | | | | | | | | | | | | | |
|  |  | | | |  | 0.002 | | | |  | 0.02 | | | |  | 4 | | | |
|  |  |  |  |  |  | us. | PMA | PMA | PMA |  | us. | PMA | PMA | PMA |  | us. | PMA | PMA | PMA |
|  |  |  |  |  |  |  |  | L-NAME | AG |  |  |  | L-NAME | AG |  |  |  | L-NAME | AG |
| Adult blood neutrophils |  |  |  |  |  | 1011  ±224 | 1626  ±221 | 1359  ±250 | 1431  ±239 |  | 1153  ±294 | 1709  ±284 | 1406  ±297 | 1466  ±296 |  | 1378  ±295 | 2062  ±216 | 1464  ±238 | 1579  ±229 |
| Cord blood neutrophils |  |  |  |  |  | 825  ±238 | 1126  ±316 | 1030  ±197 | 1002  ±267 |  | 852  ±236 | 1210  ±332 | 1104  ±203 | 992  ±212 |  | 948  ±203 | 1435  ±367 | 1320  ±328 | 1204  ±281 |
|  | Ropivacaine (mM) | | | | | | | | | | | | | | | | | | |
|  |  | | | |  | 0.0007 | | | |  | 0.007 | | | |  | 1.4 | | | |
|  |  |  |  |  |  | us. | PMA | PMA | PMA |  | us. | PMA | PMA | PMA |  | us. | PMA | PMA | PMA |
|  |  |  |  |  |  |  |  | L-NAME | AG |  |  |  | L-NAME | AG |  |  |  | L-NAME | AG |
| Adult blood neutrophils |  |  |  |  |  | 1057  ±246 | 1838  ±352 | 1498  ±335 | 1549  ±272 |  | 1064  ±211 | 1961  ±432 | 1536  ±370 | 1689  ±355 |  | 1224  ±344 | 2051  ±427 | 1648  ±390 | 1689  ±335 |
| Cord blood neutrophils |  |  |  |  |  | 936  ±215 | 1222  ±238 | 1189  ±248 | 1094  ±266 |  | 874  ±207 | 1210  ±253 | 1147  ±198 | 1017  ±244 |  | 923  ±213 | 1365  ±360 | 1268  ±251 | 1179  ±244 |

Data are the mean ± standard deviation. us., unstimulated neutrophils; PMA, phorbol 12-myristate 13-acetate; L-NAME, NG-nitro-L-arginine methyl ester; AG, aminoguanidine.

Table S4. Mean values of nitrite concentrations in the incubation media of adult (n=10) and cord blood neutrophils (n=11) exposed to LAs.

Data are the mean ± standard deviation. us., unstimulated neutrophils; PMA, phorbol 12-myristate 13-acetate.

|  |  |  | Bupivacaine (mM) | | | | | | | | |
| --- | --- | --- | --- | --- | --- | --- | --- | --- | --- | --- | --- |
|  | 0 | |  | 0.0005 | |  | 0.005 | |  | 1 | |
|  | us. | PMA |  | us. | PMA |  | us. | PMA |  | us. | PMA |
|  |  |  |  |  |  |  |  |  |  |  |  |
| Adult blood neutrophils | 0.32  ±0.085 | 0.31  ±0.083 |  | 0.30  ±0.068 | 0.31  ±0.061 |  | 0.30  ±0.070 | 0.31  ±0.071 |  | 0.30  ±0.074 | 0.30  ±0.062 |
| Cord blood neutrophils | 0.23  ±0.089 | 0.23  ±0.055 |  | 0.25  ±0.058 | 0.23  ±0.062 |  | 0.25  ±0.068 | 0.22  ±0.069 |  | 0.24  ±0.077 | 0.23  ±0.079 |
|  |  |  | Lidocaine (mM) | | | | | | | | |
|  |  |  |  | 0.002 | |  | 0.02 | |  | 4 | |
|  |  |  |  | us. | PMA |  | us. | PMA |  | us. | PMA |
|  |  |  |  |  |  |  |  |  |  |  |  |
| Adult blood neutrophils |  |  |  | 0.30  ±0.070 | 0.32  ±0.070 |  | 0.31  ±0.082 | 0.31  ±0.076 |  | 0.33  ±0.090 | 0.33  ±0.062 |
| Cord blood neutrophils |  |  |  | 0.24  ±0.073 | 0.22  ±0.073 |  | 0.24  ±0.071 | 0.23  ±0.070 |  | 0.27  ±0.070 | 0.28  ±0.080 |
|  |  |  | Ropivacaine (mM) | | | | | | | | |
|  |  |  |  | 0.0007 | |  | 0.007 | |  | 1.4 | |
|  |  |  |  | us. | PMA |  | us. | PMA |  | us. | PMA |
|  |  |  |  |  |  |  |  |  |  |  |  |
| Adult blood neutrophils |  |  |  | 0.34  ±0.12 | 0.35  ±0.11 |  | 0.33  ±0.11 | 0.35  ±0.10 |  | 0.34  ±0.11 | 0.33  ±0.071 |
| Cord blood neutrophils |  |  |  | 0.26  ±0.074 | 0.24  ±0.71 |  | 0.25  ±0.061 | 0.25  ±0.53 |  | 0.26  ±0.065 | 0.25  ±0.052 |

Table S5. Intracellular NO production in neutrophils incubated with LAs - a comparative model of multifactorial ANOVA. Main effects and interactions between the assessed factors ‘anaesthetic’ – bupivacaine, lidocaine, ropivacaine; ‘concentration category’ – concentrations of LAs categorized by their potency: the lowest, middle, the highest; ‘group’ - cord blood neutrophils, adult blood neutrophils; ‘NOS stimulation and inhibition’ – neutrophils unstimulated, PMA-stimulated, PMA-stimulated and incubated with L-NAME, PMA-stimulated and incubated with AG.

DF, degrees of freedom; DF error, the denominator degrees of freedom; F, the ratio of the factor variance to the error variance. * denotes an interaction between the factors. Significance at *P* ˂ 0.05.

|  |  | DF | DF error | | F | *P*-value |
| --- | --- | --- | --- | --- | --- | --- |
| Factors | Group | 1 | 19 | 17.255 | | 0.0005 |
|  | NOS stimulation and inhibition | 3 | 660 | 262.712 | | <0.0001 |
| Interaction | Group*NOS stimulation and inhibition | 3 | 660 | 31.254 | | <0.0001 |
|  |  |  |  |  | |  |
| Factor | Concentration category | 2 | 660 | 82.030 | | <0.0001 |
| Interactions | Group*concentration category | 2 | 660 | 2.729 | | 0.066 |
|  | Concentration category*NOS stimulation and inhibition | 6 | 660 | 0.858 | | 0.526 |
|  | Group*concentration category*NOS stimulation and inhibition | 6 | 660 | 2.056 | | 0.056 |
|  |  |  |  |  | |  |
| Factor | Anaesthetic | 2 | 660 | 46.181 | | <0.0001 |
| Interactions | Anaesthetic*concentration category | 4 | 660 | 3.238 | | 0.0121 |
|  | Anaesthetic* NOS stimulation and inhibition | 6 | 660 | 5.219 | | <0.0001 |
|  | Group*anaesthetic | 2 | 660 | 8.006 | | 0.0004 |
|  | Group*anaesthetic*concentration category | 4 | 660 | 0.672 | | 0.611 |
|  | Group*anaesthetic* NOS stimulation and inhibition | 6 | 660 | 2.910 | | 0.008 |
|  | Anaesthetic*concentration category *NOS stimulation and inhibition | 12 | 660 | 0.771 | | 0.681 |
|  | Group*anaesthetic*concentration category * NOS stimulation and inhibition | 12 | 660 | 0.457 | | 0.939 |

Table S6. Intracellular NO production in neutrophils incubated with LAs and nitrite concentrations in the incubation media – multifactorial ANOVA. Main effects and interactions between the assessed factors: ‘anaesthetic’ – bupivacaine, lidocaine, ropivacaine; ‘concentration category’ – concentrations of LAs categorized by their potency: the lowest, middle, the highest; ‘group’ - cord blood neutrophils, adult blood neutrophils; ‘NOS stimulation and inhibition’ – neutrophils unstimulated, PMA-stimulated, PMA-stimulated and incubated with L-NAME, PMA-stimulated and incubated with AG (for nitrite estimations ‘NOS stimulation’ only).

DF, degrees of freedom; DF error, the denominator degrees of freedom; F, the ratio of the factor variance to the error variance. * denotes an interaction between the factors. Significance at *P* ˂ 0.05.

|  | Intracellular NO production |  | | | |  |  | | | | |  | | | |
| --- | --- | --- | --- | --- | --- | --- | --- | --- | --- | --- | --- | --- | --- | --- | --- |
|  |  | Bupivacaine | | | |  | Lidocaine | | | | | Ropivacaine | | | |
|  |  | DF | DF error | F | *P*-value |  | DF | DF error | F | *P*-value |  | DF | DF error | F | *P*-value |
| Factors | Group | 1 | 19.01 | 15.283 | 0.0009 |  | 1 | 18.99 | 18.173 | 0.0004 |  | 1 | 18.99 | 18.081 | 0.0004 |
|  | Concentration category | 3 | 284 | 28.559 | <0.0001 |  | 3 | 282.1 | 53.303 | <0.0001 |  | 3 | 284 | 41.054 | <0.0001 |
|  | NOS stimulation and inhibition | 3 | 284 | 79.708 | <0.0001 |  | 3 | 282 | 100.197 | <0.0001 |  | 3 | 284 | 118.864 | <0.0001 |
|  |  |  |  |  |  |  |  |  |  |  |  |  |  |  |  |
| Interactions | Group*Concentration-category | 3 | 284 | 0.269 | 0.848 |  | 3 | 282.1 | 1.838 | 0.141 |  | 3 | 284 | 5.626 | 0.0009 |
|  | Group* NOS stimulation and inhibition | 3 | 284 | 9.990 | <0.0001 |  | 3 | 282 | 11.797 | <0.0001 |  | 3 | 284 | 21.070 | <0.0001 |
|  | Concentration category*NOS stimulation and inhibition | 9 | 284 | 0.942 | 0.489 |  | 9 | 282 | 2.680 | 0.005 |  | 9 | 284 | 3.581 | 0.0003 |
|  | Group*concentration-category*NOS stimulation and inhibition | 9 | 284 | 0.411 | 0.929 |  | 9 | 282 | 1.329 | 0.222 |  | 9 | 284 | 0.924 | 0.505 |
|  |  |  | | | |  |  | | | | |  | | | |
|  | Nitrite concentrations in the neutrophil incubation media |  | | | |  |  | | | | |  | | | |
| Factors | Group | 1 | 17.07 | 5.953 | 0.0259 |  | 1 | 17.08 | 5.870 | 0.0278 |  | 1 | 17.1 | 7.519 | 0.0138 |
|  | Concentration category | 3 | 113.1 | 0.085 | 0.968 |  | 3 | 113.1 | 8.860 | <0.0001 |  | 3 | 113.3 | 2.088 | 0.106 |
|  | NOS stimulation | 1 | 113.2 | 0.780 | 0.379 |  | 1 | 113.1 | 0.858 | 0.356 |  | 1 | 113.2 | 0.0126 | 0.911 |
|  |  |  |  |  |  |  |  |  |  |  |  |  |  |  |  |
| Interactions | Group*concentration category | 3 | 113.1 | 1.447 | 0.233 |  | 3 | 113.1 | 1.539 | 0.208 |  | 3 | 113.3 | 0.090 | 0.966 |
|  | Group* NOS stimulation | 1 | 113.2 | 2.615 | 0.109 |  | 1 | 113.1 | 0.478 | 0.491 |  | 1 | 113.2 | 0.253 | 0.616 |
|  | Concentration category*NOS stimulation | 3 | 113.2 | 0.125 | 0.945 |  | 3 | 113.2 | 0.045 | 0.987 |  | 3 | 113.2 | 0.521 | 0.669 |
|  | Group*concentration category*NOS stimulation | 3 | 113.2 | 0.908 | 0.440 |  | 3 | 113.2 | 1.294 | 0.280 |  | 3 | 113.2 | 0.283 | 0.839 |
